# Books and Magazines

**Published:** 1901-07

**Authors:** 


THE HOSPITALS OF JAPAN. By Edward C. Register, M. D., President of the Board of Medical Examiners State of North Carolina; Ex-President of the Charlotte Medical Society.

Dr. Register has favored us with a reprint of his article on "The Hospitals of Japan," which appeared in his journal, the *Charlotte Medical Journal,* for which he has our thanks. It is intensely interesting, and is written in the doctor's well-known easy and graceful style. The paper was written from personal observation during a recent and protracted visit to that wonderful country.

---

A SYLLABUS OF NEW REMEDIES AND THERAPEUTIC MEASURES; with Chemistry, Physical Appearance and Therapeutic Application. By J. W. Wainwright, M. D., Member of the American Medical Association; New York State Medical Association; United States Pharmacopœial Convention, 1900; American Chemical Society, Etc. Pages, 229. Price, $1.00, net. G. P. Engelhard & Co., 358-362 Dearborn St., Chicago. 1901.

THE ACUTE CONTAGIOUS DISEASES OF CHILDHOOD. By Marcus P. Hatfield, A. M., M. D., Professor Emeritus of Diseases of Children, Northwestern University Medical School; Professor of Diseases of Children, Chicago Clinical School; Attending Physician Wesley Hospital. Pages, 142. Price, $1.00, net. G. P. Engelhard & Co., 358-362 Dearborn St., Chicago. 1901.

The above books from Engelhard & Co. are neat little volumes in red cloth, neatly printed, and contain much that will be of interest and value to any practitioner. The price, $1.00, is merely nominal, and should insure a big sale.

